# Beyond Species Richness: The Importance of Phylogenetic Dimensions of Biodiversity in Disease Ecology

**DOI:** 10.1093/icb/icaf093

**Published:** 2025-06-12

**Authors:** Yingying X G Wang, Kevin D Matson, Willem Frederik de Boer

**Affiliations:** Wildlife Ecology and Conservation Group, Wageningen University & Research, Droevendaalsesteeg 3a, Wageningen 6708PB, The Netherlands; EpiCenter for Disease Dynamics, One Health Institute, School of Veterinary Medicine, University of California, Davis, Davis, CA 95616, USA; Wildlife Ecology and Conservation Group, Wageningen University & Research, Droevendaalsesteeg 3a, Wageningen 6708PB, The Netherlands; Wildlife Ecology and Conservation Group, Wageningen University & Research, Droevendaalsesteeg 3a, Wageningen 6708PB, The Netherlands

## Abstract

Biodiversity loss and anthropogenic changes to community composition have profound effects on disease emergence and transmission. These alterations influence disease risk by either amplifying or reducing disease risk, depending on the spatial scale, ecological context, and the indices used to characterize biodiversity and disease risk. We argue that species richness alone is often insufficient and that evolutionary relationships among hosts offer critical and underappreciated insights into disease dynamics. By drawing on recent empirical and theoretical studies, we highlight how phylogenetic approaches can enhance our understanding of the dynamics between biodiversity and disease. Future studies must move beyond descriptive use of phylogenetic diversity and develop mechanistic frameworks that integrate community structure and host evolutionary relationships into transmission processes to better assess disease risk under global change.

## Introduction

Emerging infectious diseases increasingly threaten global health, with most novel pathogens originating in wildlife (Wolfe et al. 2007). Many of these pathogens can infect multiple host species, creating complex transmission networks that increase the likelihood of spillover into new hosts, including humans. Meanwhile, biodiversity is decreasing rapidly due to habitat loss, land-use change, and climate change (Brooks et al. 2002; Chase et al. 2020). Changes in biodiversity have been linked to changes in disease risk (Mahon et al. 2024). Community composition can influence which pathogens are present, affect the transmission dynamics, and shape infection prevalence within host populations. Given these complexities, it is increasingly important to understand the mechanisms that link biodiversity and disease dynamics (Keesing et al. 2006, 2010).

A large body of work has linked species richness to disease outcomes, often through the concept of the dilution effect, where increased richness reduces risk by introducing non-competent hosts (Schmidt and Ostfeld 2001; LoGiudice et al. 2003; Keesing et al. 2006). This effect has been reported for a range of pathogens, including Lyme disease (LoGiudice et al. 2003), West Nile virus (Swaddle and Calos 2008), hantaviruses (Clay et al. 2009), and schistosomiasis (Johnson et al. 2009). It also applies to metazoan parasites such as branchiurans, copepods, leeches, and monogeneans, whose transmission is reduced in more diverse communities (De Baets et al. 2021). Yet, the generality of this relationship is debated (Salkeld et al. 2013; Civitello et al. 2015; Halliday et al. 2020). An evaluation of sixty-nine human parasites concluded that the dilution effect occurred in only 12% these parasites (Wood et al. 2014). Even the most well-known dilution effect example, Lyme disease, exhibits conflicting results. For example, the dilution effect appears to operate for Lyme disease in North America (e.g., LoGiudice et al. 2008; Keesing et al. 2009; Levi et al. 2016) but not in Europe (Hofmeester 2016). These inconsistencies point to the limitations of species richness as a standalone measure.

There is growing recognition that incorporating phylogenetic information is critical for understanding disease risk under biodiversity change. Biodiversity metrics that incorporate phylogenetic information can more effectively capture these shifts in community structure under disturbance than species richness alone (D'agata et al. 2014; Frishkoff et al. 2014). This is because closely related species tend to respond similarly to disturbances, as they often share key life history traits, such as body size and ecological niche (Purvis 2008; Smith et al. 2018). Consequently, species loss is typically non-random, leading to phylogenetic clustering, where communities become dominated by closely related species (Frank et al. 2017; Nowakowski et al. 2018). These related species often share physiological, immunological, or ecological characteristics that make them more likely to host the closely related pathogens (Gilbert and Webb 2007; Streicker et al. 2010; Parker et al. 2015; Mollentze et al. 2020; Toorians et al. 2024), increasing the likelihood and intensity of pathogen transmission. In ecosystems affected by habitat loss, fragmentation, or land-use change, this can increase disease risk by facilitating the spread of pathogens among related hosts.

Despite growing recognition of its importance, the integration of phylogenetic information into disease ecology has progressed slowly (Fountain-Jones et al. 2018; Filion et al. 2022). In this regard, we explore the complex relationships between biodiversity and disease in an effort to address current uncertainties and introduce phylogenetically informed perspectives. We synthesize empirical findings and theoretical developments that emphasize the importance of phylogenetic diversity for understanding disease risk under biodiversity change. Specifically, we review studies that illustrate why phylogenetic structure matters and how it can help explain the contrasting effects of species richness on disease outcomes. Furthermore, we discuss emerging frameworks for integrating phylogeny with community ecology and disease modeling. We highlight the values of moving beyond species richness and incorporating the evolutionary structure of host communities into disease ecology studies, and we call for a more mechanistic and predictive framework for anticipating and managing disease risks in a changing world.

### Is species richness a good indicator of disease risk?

**Fig. 1:**
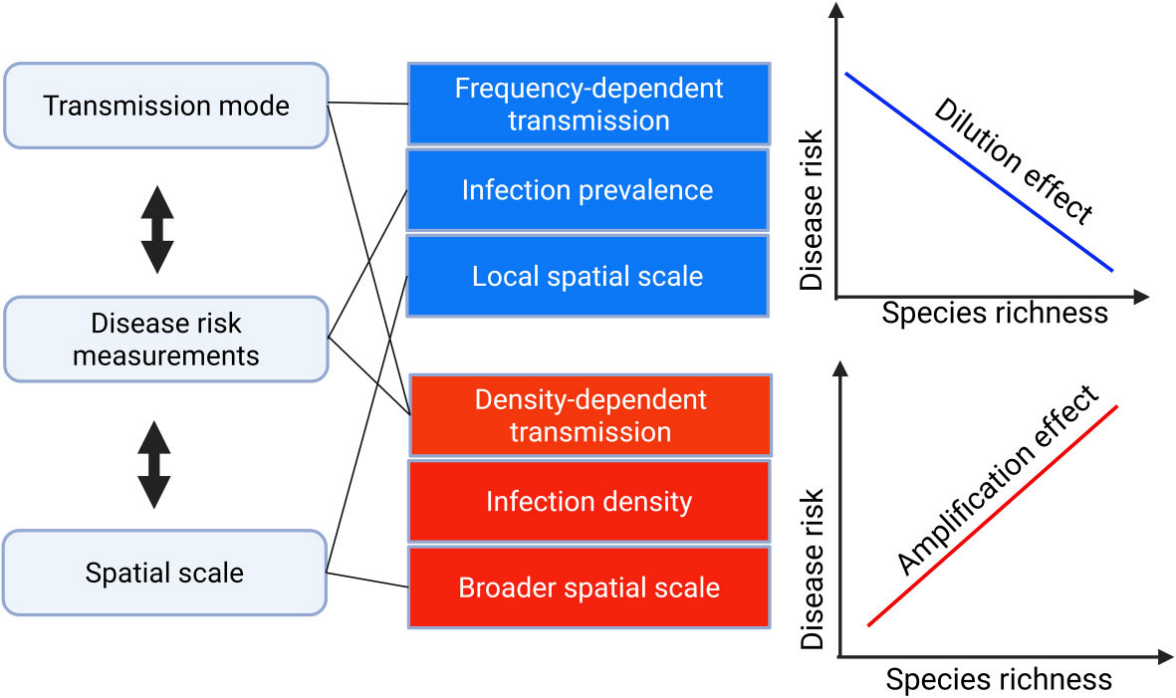
Species richness effects depend on transmission mode, disease metric, and spatial scale. Dilution effects are more frequently observed under frequency-dependent transmission, when disease is measured by infection prevalence, and at local spatial scales. In contrast, amplification effects tend to emerge under density-dependent transmission, when infection density is used as the response metric, and at broader spatial scales. Created in BioRender. Wang, Y. (2025). https://BioRender.com/kk0hyf2

Many studies of the dilution effect use species richness as a measure of biodiversity, but species richness is not always sufficient for this purpose. Three key ecological dimensions influence how richness relates to disease risk: the mode of transmission, the disease metric used, and spatial scale (Fig. 1).

The effect of species richness varies with transmission modes (Dobson 2004; Cortez and Duffy 2021). With density-dependent transmission, infection rates scale with the abundance of susceptible individuals, and adding species, especially competent ones, can increase the total abundance of susceptible individuals and amplify disease (Dobson 2004; Searle et al. 2011; Joseph et al. 2013). This amplification effect is widely observed in density-dependent diseases, such as bovine tuberculosis in badgers, where increased host density leads to higher transmission rates (Vicente et al. 2007). In contrast, frequency-dependent transmission is independent of absolute host density; transmission depends on the relative proportion of susceptible hosts in the population (Dobson 2004). This mode of transmission is often linked to dilution effects, where increasing biodiversity reduces disease risk by interrupting pathogen transmission networks (Rudolf and Antonovics 2005). Mihaljevic et al. (2014) used a combination of observational field data and mathematical modeling, found that multi-host communities disrupt pathogen transmission by reducing contact rates between competent hosts. However, theoretical models suggest that interspecific host competition and host competence can qualitatively alter these predictions (O'Regan et al. 2015; Strauss et al. 2015; Searle et al. 2016). For instance, the addition of a high-competence species may suppress transmission through strong competitive effects (O'Regan et al. 2015; Roberts and Heesterbeek 2018; Cortez and Duffy 2021).

Another key consideration is how disease risk is measured. Studies have used a range of metrics, including infection prevalence, density of infected individuals, basic reproduction number R0, and community-level R0, each of which can yield different interpretations (Roche et al. 2012; Roberts and Heesterbeek 2018; Wang et al. 2019). For instance, higher species richness has been associated with reduced overall infection prevalence but increased the total number of infected individuals due to more host individuals (Roche et al. 2012). In another study, species richness was not significantly correlated with the presence/absence of diseases but was positively correlated with disease richness (Wang et al. 2019). The latter positive relationship might be explained by the idea that “diversity begets diversity,” as more host species supply more niches for different pathogens, increasing pathogen diversity (Hechinger and Lafferty 2005; Johnson et al. 2016). Cortez (2024) shows that host community composition can affect R0, infection prevalence, and infected host density in distinct ways. First, low-competence hosts reduce R0 and prevalence but may not lower infected density if focal hosts remain abundant. Second, disease-induced mortality can shrink populations and reduce R0 while concentrating infections increases prevalence. Third, changes in focal host density from competition or mortality can lower both prevalence and density, though R0 may still rise if newly introduced hosts are efficient transmitters.

Spatial scale also influences biodiversity–disease relationships (Wood and Lafferty 2013; Kilpatrick et al. 2017). On a local scale, species interactions (e.g., those driving encounter reduction; Keesing et al. 2006) are assumed to be most influential and often favor dilution effect (Liu et al. 2023). At broad spatial scales, the consistency of the dilution effect may decline, as climate and habitat characteristics often play a more dominant role in shaping disease patterns (McGill 2010). Meta-analyses support these scale dependencies, with dilution being more common at small spatial scales (<100 km²), and amplification emerging at broader extents (>1000,000 km²; Halliday and Rohr 2019). Magnusson et al. (2020) using linear transects, reported a consistently negative relationship between species richness and disease risk across local, regional, and global scales, with no clear evidence of amplification.

Furthermore, these three dimensions (transmission dynamics, disease metrics, and spatial scale) may interact to explain the range of associations reported across biodiversity–disease studies. For example, differences in how disease risk is measured may account for the contrasting findings across spatial scales. Magnusson et al. (2020) assessed disease risk based on parasite abundance; Halliday and Rohr (2019) applied regression modeling to synthesize data from multiple studies, focusing on the overall shape of biodiversity-disease associations rather than relying on standardized parasite abundance. Additionally, transmission mode interacts with scale: locally, directly transmitted diseases (often density-dependent) may spread more easily due to close contact among hosts, whereas vector-borne, frequency-dependent diseases are more sensitive to broader-scale drivers like climate and land-use change, which affect vector populations and habitat suitability (Chala and Hamde 2021).

### Absolute biodiversity vs. change in biodiversity: what matters more for disease risk?

Disease risk is ostensibly more correlated with biodiversity change than with absolute levels of biodiversity (McCallum 2015). Halliday et al. (2020) found that biodiversity loss can increase disease risk because species that are more competent hosts for pathogens often persist or recolonize habitats more readily than less competent hosts. This shift in community composition (e.g., community disassembly) can lead to higher infection rates. Furthermore, in their meta-analysis, Halliday et al. (2020) show that loss of species after (anthropogenic) disturbances (e.g., fragmentation, deforestation, habitat loss) is more influential than natural gradients in species richness, such as those that occur in conjunction with latitude or elevation. In another meta-analysis, Mahon et al. (2024) examined how various global change drivers influence infectious disease risk, revealing that declines in established biodiversity were associated with substantially greater increases in disease outcomes compared to biodiversity variations occurring naturally, such as those along latitudinal or elevational gradients.

In fact, biodiversity and changes therein jointly determine disease risk in complex ways (Wang et al. 2021). Thus, relationships between species richness and disease risk, which are determined by the competence of the species present in the assemblage and its changes, are context-specific. Opposing effects (i.e., increases or decreases in risk) might be explained by idiosyncratic patterns of species gains and losses: disease risk increases when competent hosts are gained and decreases when incompetent hosts are gained.

### New insights into biodiversity–disease relationships

Many uncertainties surround the directions and mechanisms of relationships between disease risk and species richness. Variation in host competence and extinction vulnerability among species underscores the importance of identifying which species persist following disturbance and their roles in disease transmission. This requires extensive empirical data on species traits and host–pathogen interactions, which are often incomplete or unavailable.

Functional diversity offers a partial improvement by incorporating traits such as body size or life history (Johnson et al. 2006; Chen and Zhou 2015), but its application is constrained by challenges in trait selection and metric generalization (Cadotte et al. 2011; Hantsch et al. 2014). Phylogenetic diversity offers a more integrative framework. Because closely related species often share traits critical for pathogen transmission, phylogenetic structure can act as a proxy for both measured and unmeasured ecological traits. Phylogeny also underlies how communities assemble or disassemble under environmental change. We thus argue that phylogenetic diversity provides a more robust framework for understanding biodiversity–disease relationships than species richness alone.

### Community phylogenetic structures consistently link to disease transmission

A growing body of evidence suggests that the evolutionary structure of host communities (i.e., their phylogenetic relatedness) plays a consistent role in shaping pathogen transmission. While relatively few empirical studies have directly tested this relationship (Parker et al. 2015; Liu et al. 2016; Wang et al. 2019, French et al. 2023; Gougherty and Davies 2024), the findings across diverse systems are consistent and highlight the importance of incorporating phylogenetic structure into disease ecology.

For example, Davies and Pedersen (2008) investigated how evolutionary relationships (phylogeny) and geographical proximity influence the similarity of pathogen communities among primates, including humans. They found that closely related primate species tend to share more pathogens, suggesting that evolutionary relatedness plays a significant role in determining pathogen community composition. In another study, wildlife host communities that were phylogenetically close (i.e., small mean pairwise phylogenetic distance) were more likely to amplify disease transmission and contribute to higher disease burdens on African livestock (Wang et al. 2019). French et al. (2023) investigated viral transmission networks in an island ecosystem, finding that closely related host species share more similar viral communities. Similarly, phylogenetic relationships strongly influence host competence for avian influenza (Wille et al. 2023; Yin et al. 2023), and hence, phylogenetic diversity can predict the spatial dynamics of H5N1 outbreaks among wild birds, with more phylogenetically clustered bird communities being associated with increased disease prevalence (Huang et al. 2019). Additional evidence comes from experimental work in controlled settings. Longdon et al. (2011) infected 48 species of *Drosophila* with an RNA virus and found that viral replication success was strongly predicted by host phylogeny: closely related species supported more similar viral loads. Field-based studies have shown that phylogenetic structure remains predictive at large spatial and ecological scales. For example, in the context of Lyme disease, phylogenetic relatedness within mammal communities was positively correlated with human disease incidence at both county and state scales in the United States, but in the same study, species richness produced inconsistent, scale-dependent effects (Wang et al. 2023).

In plant communities, the phylogenetic diversity of neighboring species surrounding individual *Plantago lanceolata* plants (the focal host) strongly predicts disease severity caused by powdery mildew pathogen (*Podosphaera plantaginis*; Parker et al. 2015). In a removal experiment also involving plants, phylogenetic diversity provided the simplest and most effective predictor (e.g., better than species richness or evenness) of infection severity by a fungal pathogen (Liu et al. 2016). In forests, the evolutionary history of host tree species influences biodiversity–pest relationships: trees growing near phylogenetically distant neighbors were less likely to share pests, effectively reducing pest pressure and contributing to a dilution effect (Gougherty and Davies 2024). These empirical findings all point to the strength of biodiversity–disease relationships depending not only on species richness or diversity but also on the phylogenetic relationships within host communities.

Theoretical modeling studies further suggest that host phylogeny consistently influences disease risk across different pathogen types (e.g., bacteria, viruses) and extends to vector-borne diseases, highlighting the broad applicability of evolutionary relationships in shaping pathogen transmission dynamics. For example, Toorians et al. (2024) developed a mathematical model simulating disease transmission in multi-host communities to assess how the phylogenetic structure of host communities affects the likelihood of disease outbreaks. They found phylogenetically clustered communities (i.e., different species are closely related) amplified pathogen transmission, as related hosts often share traits that increase susceptibility to the same pathogens. Nunn et al. (2021) developed a theoretical model to assess how host extinction and vector feeding preferences influence vector-borne disease risk within phylogenetically structured host communities. Their model showed that host extinction alters the phylogenetic structure, which can either increase disease risk if distantly related, low-competence hosts are lost (leaving a more phylogenetically clustered, high-competence host community) or decrease disease risk if extinction removes closely related, high-competence hosts. Additionally, the model highlighted how vector feeding preferences can interact with host phylogeny: specialist vectors that prefer related hosts promote transmission; generalist vectors feeding on phylogenetically diverse hosts limit pathogen persistence by reducing effective transmission routes. This occurs because while generalist vectors encounter multiple host species, not all of these hosts are competent for the pathogen, reducing the likelihood of successful pathogen transmission and maintaining disease cycles within the host community.

### Why phylogenetic structure matters for disease risk?

Recent studies converge on a common insight: phylogenetic information provides a powerful tool to understand disease risk under biodiversity change (Suzán et al. 2015; Fountain-Jones et al. 2018; Filion et al. 2022). For example, Suzán et al. (2015) proposed that habitat fragmentation and species dispersal reshape community composition in non-random ways, with phylogenetic structure influencing which hosts are most likely to persist and transmit pathogens. Fountain-Jones et al. (2018) extended this idea, suggesting that environmental filtering and competitive exclusion often lead to phylogenetic clustering, which in turn facilitates pathogen sharing. Davies (2021)offered additional insight into how evolutionary history may help explain patterns of pathogen host range and how species respond to environmental changes that influence disease transmission. Filion et al. (2022) further argued that biodiversity loss is not phylogenetically random—it may selectively remove distantly related or low-competence hosts, thereby increasing transmission. Thus, the power of phylogenetic information is that it captures two fundamental processes: community assembly and pathogen transmission.

Phylogenetic structure can clarify how communities assemble and disassemble under environmental change. The structure and composition of wildlife communities, shaped by climate change and habitat loss, play a crucial role in disease transmission. Changes in community composition can alter host–pathogen interactions, potentially increasing zoonotic disease transmission (Johnson et al. 2019; Mahon et al. 2024). Therefore, understanding how community assemblages are structured and how they change when species are lost (species disassembly) is essential for unraveling the biodiversity–disease relationship and explaining why pathogen dilution occurs in some cases but not others. Integrating phylogenetics into community ecology has been recognized as a powerful tool for predicting how communities respond to global changes because it reveals the evolutionary constraints on species assembly and functional traits (Webb et al. 2002; Cavender-Bares et al. 2009). Webb et al. (2002) introduced the concepts of phylogenetic clustering and overdispersion, showing that species sorting within communities is shaped by evolutionary information and ecological interactions. Environmental filtering often results in phylogenetic clustering since closely related species tend to share functional traits that help them persist under similar conditions. In contrast, strong competition can lead to phylogenetic overdispersion by favoring distantly related species with divergent niches. Cavender-Bares et al. (2009) also highlighted that key survival traits, e.g., drought tolerance and temperature resistance, are often phylogenetically conserved, making closely related species similarly vulnerable to environmental stressors. Furthermore, phylogenetic patterns do more than serve as proxies for community assembly mechanisms; they provide deeper insights into the processes that shape ecological communities (Gerhold et al. 2015). Phylogenetic approaches integrate both observed and unmeasured traits that shape the responses of species to disturbance, interactions between species, and overall community stability. This makes these approaches especially relevant for understanding how community disassembly alters disease dynamics, as host relatedness can influence both transmission potential and susceptibility (Webb et al. 2002; Gerhold et al. 2015; Olival et al. 2017).

Phylogenetic similarity also predicts which species are likely to share pathogens. Closely related species are more likely to share pathogens, reflecting the role of phylogenetic structure in shaping pathogen transmission and host range (Gilbert and Webb 2007; Davies and Pedersen 2008; Streicker et al. 2010; Parker et al. 2015; Farrell and Davies 2019). This pattern is likely due to similarities in their evolved defenses, which are conserved within evolutionary lineages (Streicker et al. 2010). Closely related species are more biologically similar than distantly related ones (Harvey and Pagel 1991; Harvey 1996; Freckleton et al. 2002), resulting in lower immunological barriers (Pfenni 2000; Vienne et al. 2009; Longdon et al. 2011). Thus, the evolutionary distance between species is likely inversely related to the likelihood of between-species transmission and the competence of the “novel host.”

In vector-borne disease systems, host phylogeny influences vector-host contact patterns and the host preferences of vectors. From the perspective of vector-host contacts, close relatives often share ecological niche space (McCoy et al. 2013). For example, in the Lyme system, shared niches can equate to increased contact rates between suitable hosts and ticks. With ticks feeding and completing their life cycles more easily, tick abundance, and potentially disease risk, can increase. From the perspective of host preferences of vectors, phylogenetic relatedness among host species plays a significant role in shaping tick preferences, likely due to shared ecological traits among related hosts that make them more suitable for specific tick species (Esser et al. 2016).

### Phylogenetic structure can help elucidate the mechanisms behind opposing biodiversity–disease relationships

**Fig. 2:**
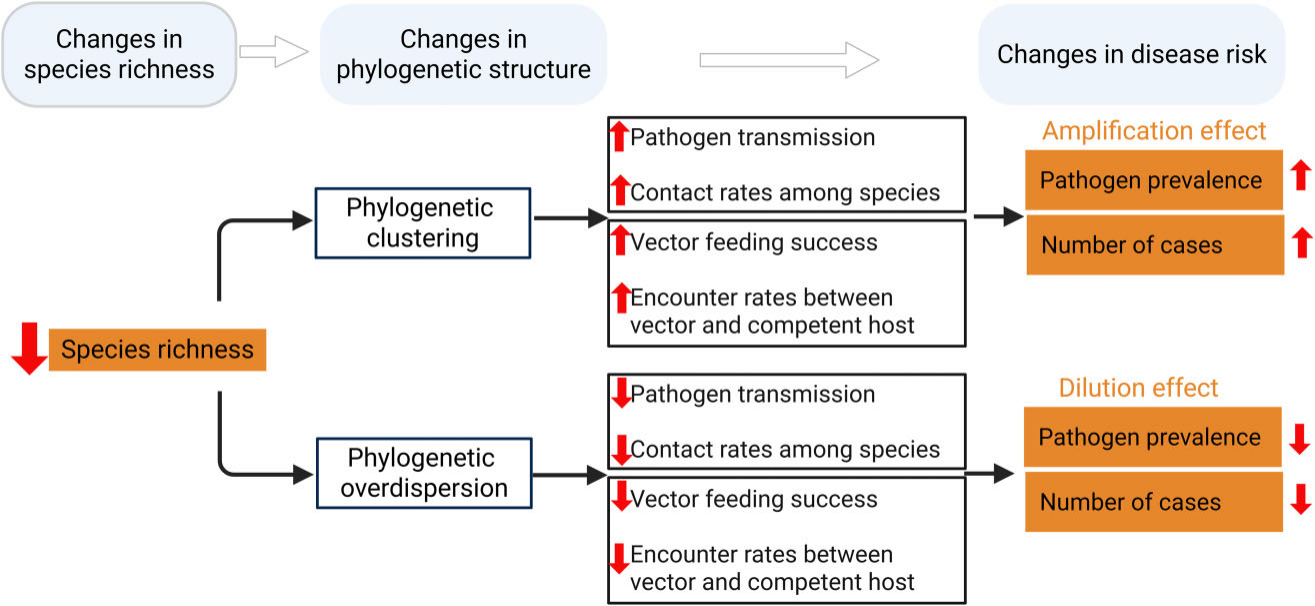
Changes in phylogenetic relatedness in a community with species loss (i.e., decreases in species richness). Decreases in species can lead to phylogenetic clustering or phylogenetic overdispersion. If phylogenetic clustering occurs, disease risk will increase because, for example, contact rates among species increase. Thus, amplification occurs. If phylogenetic overdispersion occurs when species richness is reduced, disease risk will decrease as transmission of pathogen to phylogenetically distant species is less likely to occur. Thus, dilution occurs. Created in BioRender. Wang, Y. (2025). https://BioRender.com/praop91

Studying phylogenetic structure offers valuable insights into the mechanisms driving biodiversity–disease relationships, particularly in understanding the opposing effects of species richness on pathogen dilution and amplification (Fig. 2; Fountain-Jones et al. 2018). Species loss due to environmental change often results in phylogenetic clustering, as related species often persist under similar conditions (Fig. 2). However, when competition is the dominant force, communities may instead exhibit phylogenetic overdispersion, where distantly related species are favored due to reduced niche overlap. These community assembly processes ultimately determine whether environmental change leads to phylogenetic clustering or overdispersion, influencing both ecological interactions and disease dynamics (Fig. 2, the first and second columns).

When phylogenetic clustering occurs after species loss, directly transmitted pathogens may spread more easily, as closely related species often share immunological traits (Fig. 2, the third column; Gilbert and Webb 2007; Longdon et al. 2011). Contact rates among species would also increase as those closely related species are likely to co-occur (to some extent) in the same or related habitats (McCoy et al. 2013). We expect a higher pathogen prevalence and a greater number of disease cases, leading to an overall amplification effect.

When phylogenetic overdispersion occurs after species loss, the probability of pathogen transmission is expected to decrease, as distantly related species often differ in immune function, leading to reduced cross-species transmission (Gilbert and Webb 2007; Longdon et al. 2011). Additionally, contact rates among species would decline, as phylogenetically distant species are more likely to occupy different habitats and use different resources, thereby reducing opportunities for direct transmission. As a result, we expect a lower pathogen prevalence and fewer disease cases, leading to an overall dilution effect.

For vector-borne pathogens, these transmission processes also involve vector feeding success. Closely related host species often exhibit comparable physiological characteristics, such as skin structure and immune responses, which can affect attachment and feeding success (McCoy et al. 2013). Vectors, such as ticks, often evolve preferences for particular host lineages, driven by long-term co-evolution with specific host defenses and behaviors. This co-evolution can increase host specificity, making some vectors more efficient at parasitizing hosts with which they have a deep evolutionary association, as seen in certain tick–host relationships (Esser et al. 2016). Phylogenetically related hosts frequently share similar habitats that increase the likelihood of tick-host encounters, and ticks often are abundant in habitats that align with those of their preferred hosts, enhancing their chances of successful host location and attachment.

**Fig. 3:**
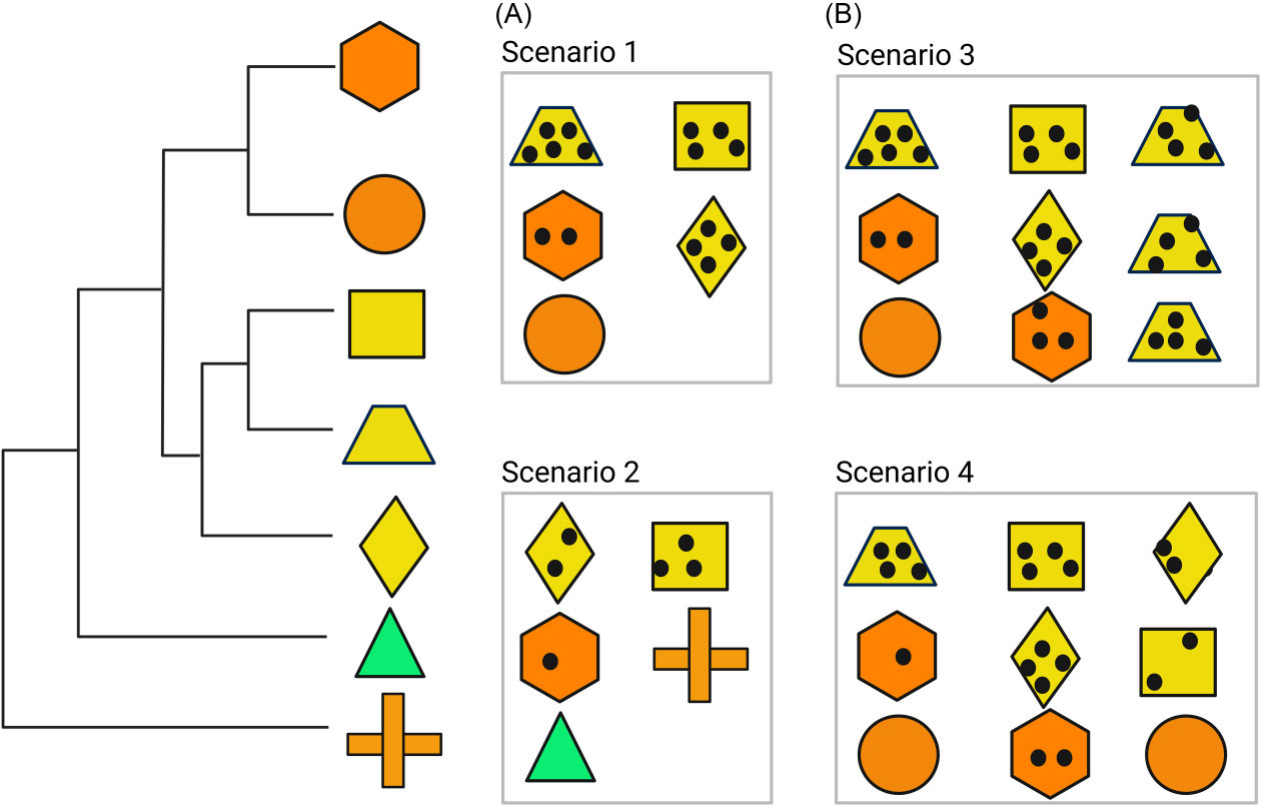
Conceptual diagram of disease risk in two hypothetical communities that have a different level of host phylogenetic relatedness. Left side: Phylogenetic tree of seven species. Shapes indicate different host species. The same color indicates that species are closely related. In (A) and (B), black spots indicate infection, and the number of black spots indicates infection intensity. (A) Scenarios 1 and 2 have the same richness (5 species), but scenario 1 is more phylogenetically clustered than scenario 2. (B) Scenarios 3 and 4 also have species richness (5 species) and the same level of host phylogenetic relatedness when abundance is not considered. When abundance is taken into account using a weighted phylogenetic relatedness value, scenario 3 is more phylogenetically clustered than scenario 4, since scenarios 3 and 4 differ in the relative abundance of species. This figure was inspired by the conceptual diagram presented in Halliday et al. (2019). Created in BioRender. Wang, Y. (2025) https://BioRender.com/ixbb48v

Understanding phylogenetic relatedness is essential for assessing disease risk, as closely related species often share similar traits that influence pathogen transmission. However, also accounting for species abundance (absolute or relative) is important, as it shapes the magnitude of each species’ contribution to transmission dynamics (Fig. 3B). A commonly used approach is to weight phylogenetic metrics by species abundance, assigning greater influence to more common species (Parker et al. 2015). This is particularly useful in assemblage analyses since species are rarely equally abundant (Webb et al. 2002; Anderson et al. 2004; Mi et al. 2012). For example, if a highly abundant species is also a competent pathogen host, the overall disease risk in the community increases. In such cases, related species dominating the community can lead to phylogenetic clustering, as the weighted phylogenetic relatedness value (MPD.Z weighted by abundance) would be lower than the unweighted value. This approach enhances the ecological realism of biodiversity–disease assessments by capturing how both relatedness and abundance jointly influence risk.

## Future directions for incorporating phylogenetic information into disease ecology

Recent theoretical and empirical advances have highlighted the potential of phylogenetic frameworks for understanding of biodiversity–disease relationships. Yet, as Fountain-Jones et al. (2018) and Filion et al. (2022) noted, the integration of phylogenetic information into disease ecology remains a slow process. To advance toward a predictive and mechanistic eco-phylogenetic framework, two key research priorities need to be addressed.

First, future studies should move beyond correlative applications of phylogenetic diversity metrics and instead use evolutionary relationships to test explicit mechanisms of disease transmission. As emphasized in Toorians et al. (2024), growing evidence indicates that phylogenetic relatedness is a key predictor of pathogen sharing, transmission likelihood, and disease severity. These patterns may reflect conserved immunological or physiological traits among closely related species that facilitate cross-species transmission (Davies and Pedersen 2008; Streicker et al. 2010; Mollentze et al. 2020). Second, a comprehensive phylogenetic framework requires coupling host and pathogen phylogenies, incorporating co-evolutionary histories, and embedding phylogenetic structure within community ecology models (Filion et al. 2022). This approach will be critical for understanding how biodiversity loss reshapes transmission networks, especially when extinctions are nonrandom and disproportionately affect functionally or phylogenetically similar species.

With growing evidence and calls for a more integrated phylogenetic framework (e.g., Fountain-Jones et al. 2018; Filion et al. 2022), disease ecology must move beyond species richness to consider the eco-evolutionary structure of communities. Integrating phylogeny with traits, abundance, host–pathogen interactions is essential for building predictive models of disease emergence and for guiding conservation and public health interventions in a rapidly changing world.

## Author contributions

Y.X.G.W. conceived the idea and led the writing of the manuscript. K.D.M. and W.F.d.B. contributed to the conceptual development, interpretation, and critical revision of the text. All authors participated in shaping the review’s structure and content and approved the final version of the manuscript.

## Data Availability

No new data were generated or analyzed in support of this research.

## Notes

From the symposium “Paddling Together: Navigating the Crosscurrents of Plant and Animal Biology to Explore Uncharted Waters in Disease Ecology” presented at the annual meeting of the Society for Integrative and Comparative Biology, January 3-7th, 2025. Designates co-first authors.
